# Brief Exposure to Sensory Cues Elicits Stimulus-Nonspecific General Sensitization in an Insect

**DOI:** 10.1371/journal.pone.0034141

**Published:** 2012-03-23

**Authors:** Sebastian Minoli, Isabella Kauer, Violaine Colson, Virginie Party, Michel Renou, Peter Anderson, Christophe Gadenne, Frédéric Marion-Poll, Sylvia Anton

**Affiliations:** 1 INRA, UMR 1272 Physiologie de l'Insecte, Versailles, France, Université Pierre et Marie Curie, Paris, France; 2 Departmento Biodiversidad y Biología Experimental, FCEyN, Universidad de Buenos Aires, Ciudad Universitaria, Buenos Aires, Argentina; 3 Department of Systems and Computational Neurobiology, MPI of Neurobiology, Martinsried, Germany; 4 INRA, UR 1037 Laboratoire de Physiologie et Génomique des Poissons, Campus de Beaulieu, Rennes, France; 5 Chemical Ecology, Department of Plant Protection Biology, Swedish University of Agricultural Sciences, Alnarp, Sweden; 6 Laboratoire Récepteurs et Canaux Ioniques Membranaires, Université d'Angers, UPRES EA 2647 USC INRA 1330, Angers, France; 7 Département Sciences de la Vie et Santé, AgroParisTech, Paris, France; Claremont Colleges, United States of America

## Abstract

The effect of repeated exposure to sensory stimuli, with or without reward is well known to induce stimulus-specific modifications of behaviour, described as different forms of learning. In recent studies we showed that a brief single pre-exposure to the female-produced sex pheromone or even a predator sound can increase the behavioural and central nervous responses to this pheromone in males of the noctuid moth *Spodoptera littoralis*. To investigate if this increase in sensitivity might be restricted to the pheromone system or is a form of general sensitization, we studied here if a brief pre-exposure to stimuli of different modalities can reciprocally change behavioural and physiological responses to olfactory and gustatory stimuli. Olfactory and gustatory pre-exposure and subsequent behavioural tests were carried out to reveal possible intra- and cross-modal effects. Attraction to pheromone, monitored with a locomotion compensator, increased after exposure to olfactory and gustatory stimuli. Behavioural responses to sucrose, investigated using the proboscis extension reflex, increased equally after pre-exposure to olfactory and gustatory cues. Pheromone-specific neurons in the brain and antennal gustatory neurons did, however, not change their sensitivity after sucrose exposure. The observed intra- and reciprocal cross-modal effects of pre-exposure may represent a new form of stimulus-nonspecific general sensitization originating from modifications at higher sensory processing levels.

## Introduction

Animals are innately sensitive to many biologically relevant cues of different sensory modalities originating from their environment. However, being able to adjust their innate behavioural responses according to their past experiences might be the key for adapting to a changing environment. Besides transitory and reversible effects on the sensory-motor systems that occur within a time frame of milliseconds to several minutes, experience can also have a profound effect on how sensory systems develop as demonstrated in vertebrates for the visual [1], [2], auditory [3], somatosensory [2] and olfactory systems [4], [5], both at the peripheral and central levels. In most cases, experience with a given sensory cue induces a long-lasting increase of responsiveness to the same stimulus, as shown *e.g.* in the model organism *Aplysia* [6]. In this marine slug, a repeatedly applied noxious stimulus elicits a facilitated siphon withdrawal reflex for up to three weeks [6].

Experience can, however, induce a change of sensitivity in two directions. Habituation, defined as the decrease in a behavioural response after repeated presentations of the same stimulus [7] is an experience-mediated plasticity through which animals may learn to filter out external information that is not relevant any more [8], [9]. The opposite effect is sensitization, in which individuals become more sensitive to a stimulus of particular interest once it is present, and increase in this way the probability of finding the stimulus source, or contrarily to avoid it in the case of a noxious stimulus [10]–[13]. Both sensitization and habituation are forms of non-associative learning, lacking a reward or a punishment. Contrarily, associative learning entails assigning a meaning for a previously neutral stimulus after the simultaneous occurrence with a reinforcement [14], [15]. Some of the underlying processes that provoke changes in sensitivity of the chemosensory system of insects have already been described [16]–[19].

Recently, we found that a brief exposure to sex pheromone could sensitize males of the noctuid moth *Spodoptera littoralis* to this pheromone long after the presentation of the stimulus. Males briefly exposed to the female pheromone gland extract or to the principal component of the female sex pheromone (*Z,E*-9,11-tetradecadienyl acetate, *Z,E*-9,11-14:OAc) were more sensitive to the same pheromone than naive males 27 h after the exposure [13], [19]. This increase of behavioural sensitivity was correlated with an increased sensitivity of antennal lobe (AL) neurons to sex pheromone within the primary olfactory centre, whereas no change was observed at the detection level as measured by electroantennogram (EAG) recordings.

We now asked if this brief exposure to the pheromone could also affect the behavioural responses and the perception of other relevant odours: *e.g.* plant odours, which are processed along different neural pathways within the olfactory system than pheromones. Moths use plant odour cues to find host plants and food sources, even if oriented responses towards plant odours are in general far less striking than towards sex pheromones. Male moths have shown attraction to flower odours in both laboratory and field experiments [20]–[22]. Although the input channels for sex pheromones and plant odours are well separated in male moths [23], at least up to the first olfactory integration centre, synergistic interactions between plant volatiles and the female pheromone have been shown, improving in some cases their orientation efficiency to find females [24]. Plant compounds thus might play a role not only for food search, but also in the context of mating.

Whereas olfaction is the main sense involved in detection of food sources and a mating partner, taste represents a key sense for the final assessment of plants and food sources. As a general pattern in phytophagous insects, the presence of sucrose is a sign of high food quality, whereas the presence of secondary compounds, such as quinine, may indicate toxicity or non-palatability [25]. The taste system of moths is quite accessible, as taste sensilla sensitive to sucrose or quinine are distributed over the mouthparts (the proboscis) and antennae, as it is the case in *Heliothis virescens* [26] and most other moths including *S. littoralis*. Upon contacting the antennae with a sucrose solution, these insects respond by extending their proboscis [27], [28] in search for food. This proboscis extension reflex (PER) stimulated by sugars, inhibited by alkaloids and modulated by internal factors such as hunger or age, has been widely studied in many insect species [29]–[32], and provides thus a simple way to estimate the sensitivity of their taste system.

Investigating the hypothesis of general sensitization, we explored in the present study if pre-exposure effects occur also intra-modally with non-pheromonal odours, intra-modally in the gustatory system and if cross-modal sensitization effects occur reciprocally between olfactory and gustatory stimuli relevant in different behavioural contexts. We pre-exposed male *S. littoralis* with different behaviourally relevant attractive and repulsive chemosensory cues and tested their behavioural response to olfactory stimuli on a locomotion compensator and to gustatory stimuli using the PER paradigm. Electrophysiological recordings from pheromone sensitive AL neurons and gustatory receptor neurons were carried out to provide an indication on the neural level from which behavioural modifications might originate.

## Results

### Olfactory responses to pheromone on the locomotion compensator

**Figure 1:**
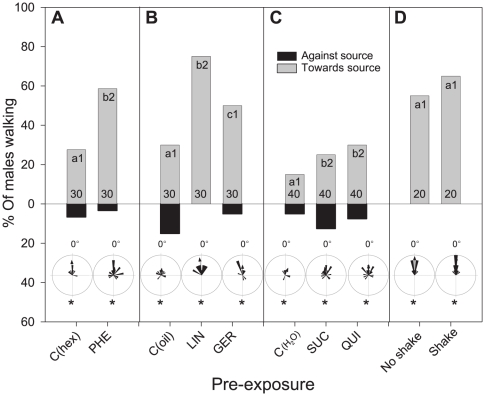
Behavioural responses to female pheromone extracts (PHE) of males pre-exposed to olfactory and gustatory stimuli on a locomotion compensator. **A**) pre-exposure to PHE, test with 0.1FE. **B**) pre-exposure to LIN and GER, test with 0.1FE. **C**) pre-exposure to SUC and QUI, test with 0.25FE. **D**) responses of males to PHE after a non-specific mechanical pre-exposure, test with 0.25FE. Grey and black columns show the percentage of males walking towards and against the source, respectively. The length of the whole column (grey+black) shows their activity level. Within each frame (A, B, C, D), locomotor activity was significantly different between columns with different lower case letters (Chi-Square, p<0.05), and orientation levels differed significantly between columns with different numbers (Chi-Square, p<0.05). Numbers at the bottom of bars indicate numbers of tested males. Circular diagrams show the mean angle of individual males (the stimulus is situated at 0°). Asterisks in circular diagrams show groups of insects that showed non-uniform distributions (Rayleigh test, p<0.05). Sensitivity to PHE was intra-modally increased by pre-exposure to PHE, LIN and GER, and cross-modally increased by pre-exposure to SUC and QUI.

When tested on a locomotion compensator with a pheromone stimulus (PHE), both naïve and pre-exposed males responded to 0.1 or 0.25 female equivalents (FE) of PHE orienting towards the source (Figure 1, Rayleigh test p<0.05 in all cases). However, olfactory (Figure 1A, B) and gustatory (Figure 1C) pre-exposure 24 h before caused a significant increase in the percentage of active males (letters in Figure 1) and males that walked mainly towards PHE (numbers in Figure 1) as compared to naïve ones. Results of a detailed statistical analysis are given below.

**Figure 2:**
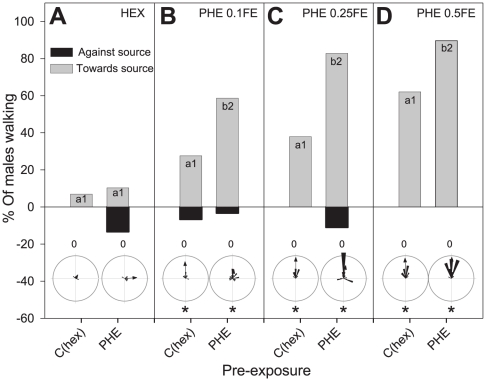
Behavioural responses to different concentrations of female pheromone extracts (PHE) of males pre-exposed to PHE on a locomotion compensator. Grey and black columns show the percentage of males walking towards and against the source, respectively. The length of the whole column (grey+black) shows their activity level. Within each frame (**A**, **B**, **C**, **D**), locomotor activity was significantly different between columns with different lower case letters (Chi-Square, p<0.05), and orientation levels differed significantly between columns with different numbers (Chi-Square, p<0.05). The number of tested males is n = 30 for each column. Circular diagrams show the mean angle of individual males (the stimulus is situated at 0°). Asterisks in circular diagrams show groups of insects that showed non-uniform distributions (Rayleigh test, p<0.05). Pheromone responses increased with the tested dose, but more pre-exposed than naïve males responded independently of the tested concentration.

As expected from previous results [13], [19], significantly more males pre-exposed to PHE (1FE) were activated (Figure 1A, *X* *^2^* = 7.63, d.f. = 1, p<0.01) and walked mainly towards 0.1FE PHE (*X^2^* = 7.21, d.f. = 1, p<0.01) as compared to the control group, C_(hex)_. Moreover, both activity and orientation levels increased with increasing PHE concentrations in control and pre-exposed males (Figure 2, positive dose-response curves) and significant differences between pre-exposed and control-exposed males were observed for all three doses of PHE (0.1, 0.25 and 0.5 FE): in all cases, responses of pre-exposed males were higher than in naïve males.

**Figure 3:**
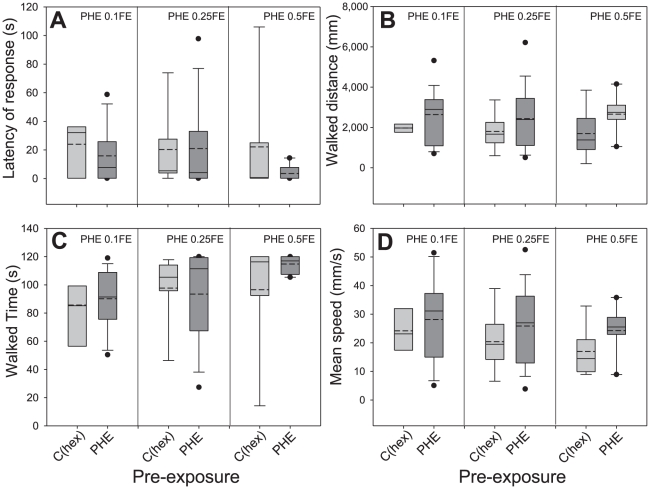
Trajectometry analyses of the walking pathways of PHE pre-exposed males confronted with different concentrations of PHE. Kinetic parameters describing different characteristics of the movement of the males on the locomotion compensator were calculated: **A**) “latency” (time until males activated), **B**) “walked distance” (displacement in any direction), **C**) “walked time” (active time) and **D**) “mean speed” (“walked distance” divided by “walked time”). The numbers of analyzed trajectories are n = 7 and n = 19 for C(hex) and PHE 0.1FE; n = 18 and n = 25 for C(hex) and PHE 0.25FE; n = 18 and n = 26 for C(hex) and PHE 0.5FE, respectively. The boundaries of the box indicate the 25th and 75th percentiles, whiskers indicate the 90th and 10th percentiles and black dots show outliers. Full and dashed lines within the box mark the median and mean respectively. Median tests (p<0.05) were carried out to reveal differences between different tested concentrations and pre-exposures. Only “walked distance” and “mean speed” increased in PHE pre-exposed male moths, but were not dependent on pheromone concentration. “Latency” and “walked time” changed with pheromone concentration but not after pre-exposure.

In addition to the differences in the percentage of males activated and oriented to the source, we analyzed if active males changed their locomotion behaviour while approaching the PHE after PHE pre-exposure. Our detailed analysis of walking tracks (Figure 3) showed that neither the latency of response (*X^2^* = 0.42, d.f. = 1, p = 0.51) nor the total walked time (*X^2^* = 0.014, d.f. = 1, p = 0.90) changed with pre-exposure, but changed significantly with the tested PHE dose (*X^2^* = 10.23, d.f. = 2, p<0.01 and *X^2^* = 15.67, d.f. = 2, p<0.001, respectively). On the other hand, the walked distance (*X^2^* = 13.71, d.f. = 1, p<0.001) and the mean walking speed (*X^2^* = 10.99, d.f. = 1, p<0.001) were significantly higher in PHE pre-exposed males than in control males and did not change with doses (*X^2^* = 3.11, d.f. = 2, p = 0.21 and *X^2^* = 1.87, d.f. = 2, p = 0.39, respectively). This result shows that pre-exposed males not only respond more frequently to the pheromone, but that responding males also improve their oriented locomotion behaviour (*i.e.* they walk faster towards the source) as compared to control males.

**Figure 4:**
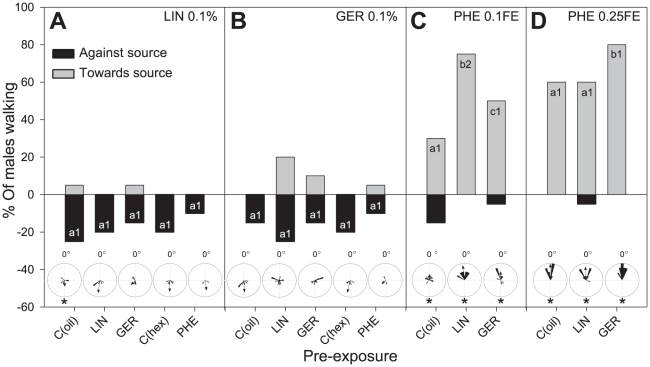
Behavioural responses to linalool (A: LIN), geraniol (B: GER) and different concentrations of female pheromone extracts (C and D: PHE) of males pre-exposed to LIN, GER and PHE. Grey and black columns show the percentage of males walking towards and against the source, respectively. The length of the whole column (grey+black) shows their activity level. Within each frame (A, B, C, D), the percentage of PER responses were significantly different between columns with different letters (Chi-Square, p<0.05). The number of tested males is n = 30 for each column. Circular diagrams show the mean angle of individual males (the stimulus is situated at 0°). Asterisks in circular diagrams show groups of insects that showed non-uniform distributions (Rayleigh test, p<0.05). Sensitivity to LIN and GER did not vary with any kind of pre-exposure. Sensitivity to PHE increased with both LIN and GER pre-exposure.

Males did not respond clearly to stimulation with 0.1% linalool (LIN) or geraniol (GER) (Figure 4A, B). Pre-exposure to LIN and GER (0.1%) modified, however, the response level of males to 0.1FE PHE as compared to naïve males, C_(oil)_ (Figure 1B; Figure 4C, D). Pre-exposure to LIN significantly increased the activity and orientation of males to PHE (*X^2^* = 7.67, d.f. = 2 and *X^2^* = 6.12, d.f. = 2, respectively, p<0.05 in both cases). Pre-exposure to GER did not modify the activity level of males (*X^2^* = 4.82, d.f. = 2, p = 0.09) but increased their oriented response (*X^2^* = 6.98, d.f. = 2, p<0.05).

Gustatory pre-exposure by touching the antennae with 1 M sucrose (SUC) or 0.1 M quinine (QUI) (Figure 1C) significantly increased the activity (*X^2^* = 7.20, d.f. = 2 and *X^2^* = 6.83, d.f. = 2, respectively, p<0.05 in both cases) and orientation (*X^2^* = 7.12, d.f. = 2 and *X^2^* = 6.98, d.f. = 2, respectively, p<0.05 in both cases) of males towards the pheromone source on the locomotion compensator as compared to control-exposed males, C(H_2_O). However, these differences were relatively small possibly due to the much lower absolute response rates to PHE males exhibited during this experimental series as compared to other series (*e.g.* Figure 1A, B; Figure 2).

To test if only pre-exposure to stimuli relevant to resource location causes changes in pheromone responses, or if contrarily, experience of a new context was sufficient to elicit a similar increased sensitivity, a non-specific mechanical stimulus was generated by shaking males for 1 minute on a laboratory shaker. No changes in the behaviour of shaken males caused by this non-specific pre-exposure was found (Figure 1D, for activity *X^2^* = 1.72, d.f. = 1, p<0.05; for orientation *X^2^* = 1.18, d.f. = 1, p<0.05) when comparing with non-shaken males. During shaking males were highly active inside the box. Preliminary assays revealed that this activation lasted for a few minutes and that males were not physically damaged by this treatment.

### Responses of AL neurons to PHE after SUC pre-exposure

**Figure 5:**
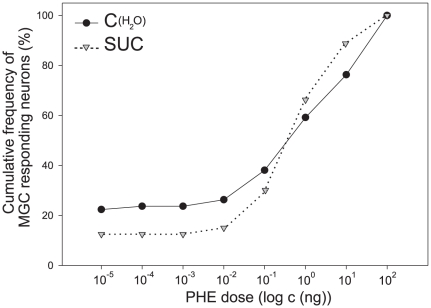
Cumulative frequency curves of response thresholds for the main pheromone compound (*Z,E*-9,11-14:OAc) of intracellularly-recorded MGC neurons in naïve (C_(H20)_) and pre-exposed (SUC) males. No significant differences between the sensitivity of naïve and pre-exposed males were found, as revealed by a G-Test comparing the percentages of AL neurons responding at different thresholds. Note the bimodal distribution of thresholds in both groups: only few neurons with an intermediate threshold were found (flat curve between 10^−5^ and 10^−2^ ng doses).

Intracellular recordings were done from 76 neurons in 35 control males (exposed to distilled water) and from 80 neurons in 40 sucrose-exposed males, penetrated within the male-specific macroglomerular complex (MGC) of the AL, dedicated to pheromone processing. In both groups, a sigmoidal curve of the cumulative frequency of responding neurons to the main pheromone component was found (Figure 5) with response thresholds ranging between 10^−5^ and 10^2^ ng. Around 20% of very sensitive neurons, a few neurons with intermediate sensitivity and high proportions of neurons with thresholds at 1 ng or higher were found. The distribution of neurons with different thresholds was not statistically different between naïve and sucrose-exposed males for any of the tested doses (G-Test, G_(5)_ = 9.9, p = 0.08).

### Gustatory responses to SUC using the PER paradigm

**Figure 6:**
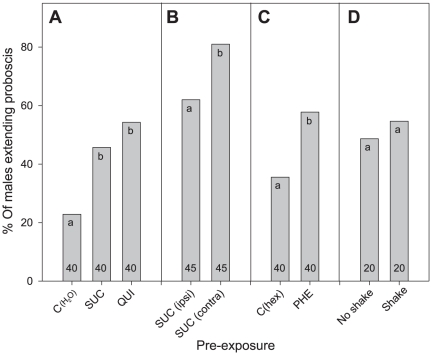
Proboscis extension reflex (PER) responses to sucrose (SUC) of males pre-exposed to gustatory and olfactory stimuli. **A**) pre-exposure to SUC and QUI, test with 0.03 M SUC. **B**) pre-exposure to SUC ipsi- or contralateral antenna, test with 0.03 M SUC. **C**) pre-exposure to PHE, test with 0.03 M SUC. **D**) responses of males to SUC after a non-specific mechanical stimulus, test with 0.1 M SUC. Columns show the percentage of males extending the proboscis when one of their antennae was contacted with a toothpick soaked with a SUC solution. Within each frame (A, B, C, D), the percentage of PER responses were significantly different between columns with different letters (Chi-Square, p<0.05). Numbers at the bottom of bars indicate numbers of tested males. Sensitivity to SUC was intra-modally increased by pre-exposure to SUC and QUI, and cross-modally increased by pre-exposure to PHE.

**Figure 7:**
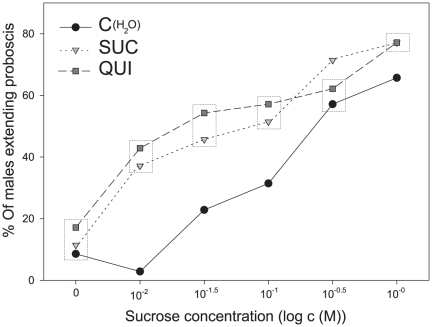
Proboscis extension reflex (PER) responses to different test-concentrations of sucrose (SUC) of males pre-exposed to sucrose (1 M SUC) and quinine (0.1 M QUI). Each point represents the percentage of males extending their proboscis when their antennae were contacted with different concentrations of SUC. The number of tested males is n = 40 for each data point. Dashed boxes enclose values that do not differ significantly (Chi-Square tests, p<0.05). Sensitivity to SUC was higher in males pre-exposed to SUC and QUI, showing a stronger difference with naïve males at low doses.

To test if also gustatory sensitivity could be altered by a brief exposure to tastants, we took advantage of the innate response of males, which extend their proboscis in a dose-dependent manner when their antennae are touched with a sucrose solution. We found this response to be modulated by a gustatory pre-exposure to SUC or QUI 24 h before testing, as more pre-exposed (SUC or QUI) than naive males (C(H_2_O)) extended their proboscis as response to the contact of 0.03 M SUC (Figure 6A; X^2^ = 7.87, d.f. = 2 and X^2^ = 8.98, d.f. = 2, respectively, p<0.05 in both cases). Similar effects of pre-exposure were found along a dose-response curve and were stronger when low doses of SUC were tested (Figure 7).

Moreover, if the pre-exposure and test were done on different antennae of the same individual (contra-lateral), the effect of pre-exposure was even stronger than if pre-exposure and test were done on the same antenna (ipsi-lateral) (Figure 6B; *X^2^* = 4.23, d.f. = 1, p<0.05).

When males were pre-exposed to PHE on the locomotion compensator before gustatory tests, a significant increase in the PER response to 0.03 M SUC was observed as compared to naïve males (Figure 6C; *X^2^* = 6.93, d.f. = 1, p<0.01).

No effect of a non-specific stress generating pre-exposure (shaking) on the gustatory response of males to 0.1 M SUC was found (Figure 6D; *X^2^* = 1.23, d.f. = 1, p = 0.35).

Different concentrations of SUC were tested along experiments because motivation of males to extend their proboscis when their antennae were stimulated with SUC solutions depended on time of the year. However, tested doses were always in the lower part of the dose-response curve, where differences between pre-exposed and control males were obvious (Figure 7).

### Responses of gustatory receptor neurons to SUC after SUC and QUI pre-exposure

**Figure 8:**
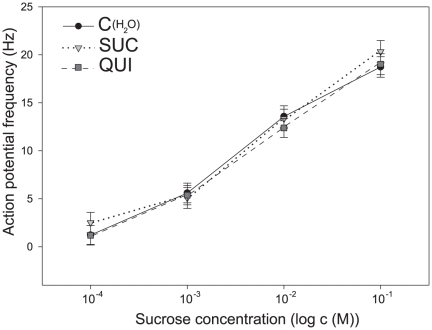
Responses of single gustatory sensilla to sucrose (SUC) in males pre-exposed to gustatory (SUC and QUI) stimuli. Each point represents the mean action potential frequency during the first second of stimulation and its standard error. Whereas responses of gustatory sensilla were dose-dependent, they did not differ statistically between pre-exposed and naïve males as revealed by a two-way ANOVA with Repeated Measures on one factor (stimulus concentration, see text).

Extracellular recordings from 287 antennal *Sensilla chaetica* in 72 male moths showed dose-dependent responses (Figure 8). The average action potential frequency of gustatory receptor neurons during the first second of stimulation, increased significantly with stimulus intensity (F = 280.63, d.f. = 3, p<0.001). Post-hoc Tukey HSD comparisons showed significant differences between the responses to the 4 doses of SUC tested (p<0.05 in all cases).

Contrarily, no effect of pre-exposure was observed (F = 0.38, d.f. = 2, p = 0.68); responses in SUC-exposed and QUI-exposed males did not differ significantly in their spike frequency nor when compared with responses in naïve males. The analysis of 100 ms bins of action potentials to evaluate the initial phasic and the following tonic part of the responses separately did not reveal any differences in firing rates between pre-exposed and naïve males either.

## Discussion

In the present study we show that a brief pre-exposure to different olfactory and gustatory stimuli elicits behavioural intra- and reciprocal cross-modal long-term sensitization in a male moth. Interestingly, although the sensitivity of AL neurons to pheromone was recently shown to change after pheromone and bat sound pre-exposure [19], [33], our new data allow us to exclude early processing stages (the antennal and AL level) to be at the origin of gustatory-evoked pheromone sensitization.

### Effects of brief odour exposure on locomotion responses to odours

Although moths generally orient to pheromone sources mainly by flying, *S. littoralis* males showed clear walking orientation on the locomotion compensator, as observed before in other moth species such as *Bombyx mori* [34], [35]. Our observations confirm the increase of attraction to the sex pheromone exhibited by *S. littoralis* males in both wind tunnel and olfactometer bioassays, 24 h after a brief pre-exposure to the pheromone [13], [19], [33]. In addition to a higher percentage of males orienting towards the pheromone source after pre-exposure, we have now evidence that pre-exposed males are not only more prone to respond to the pheromone but also walked more rapidly towards the stimulus than naïve males, whereas their response latency did not change.

Although pre-exposure with linalool or geraniol increased subsequent responses to the sex pheromone, no changes in the responses to the plant compounds themselves were found. Taking into consideration that pre-exposure to suboptimal pheromone stimuli had a much lower effect on subsequent pheromone responses than pre-exposure to a natural stimulus [13], it is possible that the doses used here for pre-exposure with plant odours were sufficient to show an effect on pheromone responses in most cases, but too low to have an effect on subsequent responses to plant odours. Another explanation could be that only single plant compounds were used in this study. As for pheromone, it is well known that insects show better responses to blends of plant odours than to single compounds [36]. Here pre-exposure to single plant odours had a positive effect on the response to the main component of the pheromone, but no effect on responses to these plant odours themselves. Possibly, pre-exposure is not sufficient to change a non-attractive stimulus into an attractive one, but can modulate the sensitivity to an attractive stimulus (the pheromone blend).

### Effects of brief gustatory exposure on proboscis extension responses to sucrose and its neurobiological basis

Proboscis extension responses to sucrose, especially at low doses, occurred more frequently in pre-exposed *S. littoralis* than in control males. The effect of pre-exposure to both sucrose and quinine on sucrose responses was highly similar, in spite of quinine being a feeding deterrent (i.e. an aversive signal) in phytophagous insects [26], [37]–[39], whereas sucrose is an appetitive stimulus. To our knowledge, this is the first time that a brief exposure to tastants is shown to increase behavioural responses to the same or even a different chemical stimulus after one whole day. In other insects, brief exposure to sucrose elicited short-term sensitization as shown by PER responses in honey bees [40] and fruitflies [7]. In infant rats, pre-exposure to tastants (quinine, sucrose) has been shown to enhance responsiveness to these substances, discussed as a form of taste familiarity [41]. Interestingly, pre-exposure was repeated many times in this latter study, while a single pre-exposure was sufficient in ours. In humans, a repeated or even a single exposure to fructose caused a sensitization to glucose, which lasted several days [42].

Our electrophysiological characterization of sucrose-sensitive antennal sensilla revealed no statistical differences in dose-response curves between sensilla of males pre-exposed to sucrose, quinine and water (control). These results are in agreement with data obtained by electroantennogram recordings in *S. littoralis* with sex pheromone stimulation, which did not reveal differences after brief pre-exposure [19]. Given these observations, we can exclude the peripheral level as a major origin of modulation of behavioural gustatory sensitivity after pre-exposure to sucrose and quinine.

### Cross-modal effects of pre-exposure and their neurobiological basis

In our behavioural experiments we revealed reciprocal cross-modal effects of pre-exposure between olfactory and gustatory stimuli. Both oriented sex pheromone responses and PER responses to sucrose improved not only after pre-exposure to stimuli of the same modality, but also after pre-exposure to the respective other modality. The idea of a general multimodal sensitization presented here was already suggested in a previous study [33], where it was shown that a behaviourally meaningful auditory stimulus can modify the sensitivity of *S. littoralis* males to the sex pheromone. Twenty-four hours after hearing an artificial bat sound (i.e. a natural predator sound) the behavioural response threshold of males to the female-emitted pheromone was lower than in naïve males. In our study, we extend this paradigm and show that the olfactory responses of males to pheromone were also cross-modally improved after an experience with both an attractive and an aversive gustatory stimulus (sucrose and quinine). Furthermore, we show that also gustatory sensitivity to sucrose improved after pre-exposure to tastants and to pheromone. These results support the hypothesis that meaningful sensory stimuli, i.e. aversive or attractive signals, might contribute to a general sensitization of different sensory modalities. Whereas previous studies have focused on intra-and cross-modal effects of brief pre-exposure on pheromone responses [19], [33], we show here for the first time that brief pre-exposure with an olfactory signal also modifies responses to gustatory stimuli. On the other hand mechanical shaking, a stimulus without a precise biological significance to the moth, did not elicit increased sensitivity to olfactory and gustatory stimuli. Biologically meaningful stimuli of the natural environment thus seem to prepare the nervous system for future encounters with the same or other environmental signals to improve behavioural responses.

Although olfactory and gustatory input reaches the brain via the same antennal nerve, their neurons target different zones. Modulatory neurons are interconnecting different brain areas, including the antennal lobe, the antennal mechanosensory and motor centre, the tritocerebrum and the subesophageal ganglion, representing the different target zones of olfactory and gustatory receptor neurons [43], [44]. An interaction of olfactory and gustatory stimuli could thus occur within any of these neural structures accounting for the cross-modal effects of pre-exposure. However, in our work, no changes of sensitivity to the pheromone were found in MGC neurons when pre-exposing males to sucrose. These results suggest that the neuronal basis of the behavioural “cross-modal” effect of gustatory pre-exposure on olfactory responses is not located at the AL level but most likely at higher brain levels (*e.g.* mushroom bodies, etc), as opposed to previously described strong effects of acoustic pre-exposure on the sensitivity of AL neurons to both sex pheromone and plant odours [33].

### Conclusions

Our results on cross-modal effects of brief pre-exposure, together with another study [33] show clearly that the observed pre-exposure effects are not a case of selective attention, but rather support the hypothesis of general sensitization by different meaningful sensory inputs. We propose that behaviourally relevant stimuli occurring in the environment might contribute to a maturation process increasing the sensitivity of olfactory, gustatory and possibly other sensory systems in moths. Sensory inputs elicit developmental processes in the nervous system of insects, including the modulation of sensory systems. Most likely, any behaviourally relevant sensory stimulus might contribute to physiological or anatomical changes, leading ultimately to increasing sensitivity inducing changes in behaviour. Since increasing the sensitivity of sensory neurons is energetically costly [45], the existence of a low/high sensitivity switch that may allow insects to economize energy by being less sensitive when there is no need to be (i.e. when no cues are present) and reactive when biologically relevant cues are present in the environment, might be biologically meaningful.

Pre-exposure effects obtained in the present study could be compared with pre-exposure effects during spatial learning [46]. In vertebrates, a cognitive map is thought to be a highly flexible representation of space that automatically updates whenever novel information appears in a known environment. Although the establishment of cognitive maps in vertebrates have been shown in paradigms using reinforcement, mere exposure to sensory cues might be sufficient to acquire information about the environment. Once a map of spatial relationships between cues within the environment has been established, an animal might use short-cuts even through unexplored areas [47]–[49]. In our model, *S. littoralis*, the brain might acquire a sort of cognitive map of its sensory environment through pre-exposure to different behaviourally relevant stimuli (*i.e.* odours of sex and food, sound of a predator, appetitive or repulsive tastants), which improves behavioural responses to the same or different signals in order to optimize survival and reproduction.

## Materials and Methods

### Insects

*S. littoralis* males and females were kept in separate climatic chambers (16L:8D, 22°C and 70–90% relative humidity) from the pupal stage. Emerged adults were kept without feeding. For behavioural olfactory tests, wings of adult males were cut at the day of emergence (day-0). Olfactory or gustatory pre-exposure was performed 2–5 h into the scotophase at day-1. Behavioural and electrophysiological tests were carried out 2–5 h into the scotophase at day-2, *i.e.* at least 24 h after pre-exposure.

### Olfactory stimuli

Pheromone extracts (PHE) were prepared from 2/3-day-old females as described previously [33]. Dilutions of 0.1FE (female equivalent), 0.25FE or 1FE were used for stimulation. For electrophysiological recordings, the main sex pheromone component ((Z,E)-9,11-tetradecadienyl acetate; >97% purity, synthesized in Versailles by Martine Lettere) diluted in hexane (HEX) was used at doses from 0.01 pg to 100 ng.

The synthetic plant-related odours linalool (LIN; Sigma, 97% purity, Saint-Quentin Fallavier, France) and geraniol (GER; Sigma, 96% purity, Saint-Quentin Fallavier, France) were used at the dose of 0.1% v/v diluted in mineral oil (MO) (Sigma, Saint-Quentin Fallavier, France). This dose was chosen because preliminary data using different doses of both compounds showed no differences in eliciting behavioural responses of males, and because it is biologically active in males of *S. littoralis* [50].

### Gustatory stimuli

Aqueous solutions of 10^−2^, 10^−1.5^, 10^−1^, 10^−0.5^ and 1 M sucrose (SUC; >99.5% purity, Sigma, Saint-Quentin Fallavier, France) and 0.1 M quinine (QUI;  = 98.0% purity, Sigma, Saint-Quentin Fallavier, France) were prepared every day. These concentrations are well detected by gustatory receptor neurons on the antennae of *S. littoralis* [51].

### Pre-exposure procedures

Olfactory pre-exposure was carried out on the locomotion compensator (see below). Males, placed 2 h prior to pre-exposure in the experimental room, were individually pre-exposed during 1 min either to 1FE of PHE, to 0.1% LIN or to 0.1% GER, under red light. Two control groups were exposed to HEX or to MO. Behaviour in response to the stimulus was monitored, and males were carefully returned to the rearing chamber until the behavioural tests or electrophysiological recordings were carried out.

Gustatory pre-exposure was done by using the proboscis extension reflex (PER). Antennal contact with a sugar solution elicits extension of the proboscis in *S. littoralis* [27], which can be used to determine the detection threshold for a sugar solution. Males were inserted in cut Pipette tips with the head protruding. Individual pre-exposure was carried out by moving a tooth pick soaked with either distilled water (H_2_O, control), 1 M SUC or 0.1 M QUI evenly over one antenna for 10 s to make sure that a maximum number of gustatory sensilla got in contact with the solution. PER responses were monitored, and males were returned to the rearing chamber until the behavioural tests or electrophysiological recordings were carried out.

In an additional series of experiments, we provided a non-specific mechanical stimulation by shaking 20 males together in a plastic box on a laboratory vortex mixer (2400 rpm) during 1 min, without application of chemical stimuli. A control group was handled without shaking in parallel. Males were then returned to the rearing chamber until the olfactory or gustatory tests were carried out.

### Behavioural olfactory tests

A locomotion compensator (LC-300, Syntech, Kirchzarten, Germany) was used to analyze the olfactory behaviour of males in presence of a PHE source as described previously [52]. Briefly, wingless males were released at the top of the plastic sphere (30 cm Ø), which is rotated opposite to the insect displacement by motors controlled by a camera located above the insect, while the insect is maintained in a constant position. Rotational movements are transferred to a computer, and incremented as X and Y coordinates in 0.1 s intervals from which the trajectory of each insect was reconstructed. Males were exposed to a constant charcoal filtered humidified airflow (17 ml/s). An additional airflow (7 ml/s) was switched to a parallel tube with the pipette or vial containing the odorant, using a stimulus controller (CS 55, Syntech, Kirchzarten, Germany). A pipette, containing HEX on a filter paper or a vial containing 1 ml of MO was used as control.

Naïve or pre-exposed males were placed in the experimental room before the onset of scotophase. At the time of the experiment, males were individually placed on the locomotion compensator, left for 1 min for acclimatization, and their behaviour was recorded in response to PHE during 2 min. The following experimental series were performed:

pre-exposure to PHE, test with PHE
pre-exposure to LIN or GER, test with PHE
pre-exposure to SUC or QUI, test with PHE
pre-exposure to shaking, test with PHE

Different pheromone doses were used in different experiments because “absolute” response thresholds to PHE varied between seasons. A sub-optimal pheromone dose depending on the “absolute” threshold during a given test-period was chosen to reveal possible differences between pre-exposed and naive males for each experimental series.

The existence of a preferred direction of males in relation to the olfactory stimulus was analyzed using circular statistics and graphs (Oriana software, Kovach Computing Services, Anglesey, Wales). The position of the air stream outlet was designated at 0°. The statistical evidence of a uniform distribution around a circle was tested using the Rayleigh test [53].

The locomotor behaviour of males was analyzed using the TrackSphere 3.1 software (Syntech, Kirchzarten, Germany) and the following parameters were calculated for individual trajectories:

Upward length is the net displacement towards the stimulus in mm. Positive or negative values denote an overall preference to walk towards or against the stimulus, respectively.

Effects of pre-exposure were assessed by comparing the percentages of active males (males moving towards plus males moving against the stimulus) between treatments. Changes in the oriented response of males caused by pre-exposure were analyzed by comparing the proportion of males walking “towards” or “away from” the stimulus source between treatments. Chi-square tests for homogeneity were performed for comparing percentages of activated or oriented males of different groups.

Differences in individual trajectories were analyzed and compared between treatments by calculating 4 kinetic parameters:

latency of response (s): time until males activated (non-stop walk for at least 100 mm)
walked distance (mm): displacement in any direction
walked time (s): active time within the total assay duration
mean speed (mm/s): “walked distance” divided by “walked time”

Non-parametrical comparisons between these kinetic parameters of naïve and pre-exposed males and changes in parameters associated to the tested PHE dose were assessed using the Median test.

### Behavioural gustatory tests

Gustatory tests were done by using the PER paradigm. A toothpick soaked with different aqueous solutions was moved evenly over the antenna during 10 s to make sure that a maximum number of gustatory sensilla got in contact with the solution and the occurrence of a PER was registered. The following experimental series were performed:

pre-exposure to SUC or QUI, test with SUC
pre-exposure to SUC, test with SUC on the ipsi- or contra-lateral antenna
pre-exposure to PHE, test with SUC
pre-exposure to shaking, test with SUC

Different concentrations of SUC were used in different experiments because “absolute” response thresholds to SUC varied between time periods. As for pheromone tests, a sub-optimal sucrose concentration depending on the “absolute” threshold during a given test-period was chosen to reveal possible differences between pre-exposed and naïve males for each experimental series. The proportion of males extending the proboscis was calculated for each group and compared by means of a Chi-Square test for homogeneity.

### Intracellular recordings from AL neurons

Pre-exposed males were mounted and prepared for electrophysiology as previously described [19]. Intracellular recordings of pheromone sensitive neurons within the MGC were performed using standard recording techniques [54]. When intracellular contact had been established, a 500 ms pheromone stimulus was introduced into a constant air stream (7 ml/s) using a stimulus controller (CS 55, Syntech, Kirchzarten, Germany). A minimum of 10 s elapsed between individual pheromone stimulations. Recorded signals were amplified (Axoclamp-2B, Axon Instruments, Foster City, USA), digitalized and analyzed with Autospike 32 software (Syntech, Kirchzarten, Germany).

We counted the number of action potentials during the excitatory response period of the neuron and subtracted the spontaneous activity during the same duration of time before the stimulus onset. A neuron was classified as responding to the different concentrations of the pheromone when the response exceeded the response to a control stimulus (the solvent hexane) by at least 10%. The percentage of AL neurons responding at different thresholds was calculated and compared between naïve and pre-exposed males with a G-Test (applying Williams correction).

### Extracellular recordings from antennal gustatory sensilla

Neural activity of gustatory receptor neurons was studied using the tip recording technique [55]. Insects were fixed in tight-fitting plastic tubes with their head and antennae protruding. The glass capillary containing the stimulus, and an electrolyte (1 mM KCl) was fitted onto a silver wire connected to the probe of a preamplifier and brought in contact with the tip of a *Sensillum chaeticum* (TastePROBE DTP-02, Syntech, Kirchzarten, Germany). Data acquisition and storage was triggered by voltage signals when contact between the sensillum and the stimulus solution was established. Electrical signals were further amplified (CyberAmp 320, Axon Instruments, Foster City, USA) and filtered (band-pass 10–2800 Hz). Responses were recorded during 2 s and the recording electrode was immediately removed from the sensillum tip after each recording. At least 10 s elapsed between individual stimuli. Spikes were detected and counted using custom dbWave software [56]. The average number of spikes during the first second of stimulation was calculated for each stimulus concentration and separately for control, SUC- and QUI-treated males. Dose-response curves for the different treatments were compared by a Two-way ANOVA with Repeated Measures on one factor (stimulus concentration). Tukey post-hoc comparisons were performed to compare spike frequencies of pre-exposed and naïve males.

## References

[1] Crair MC, Gillespie DC, Stryker MP (1998). The role of visual experience in the development of columns in cat visual cortex.. Science.

[2] Fox K, Wong ROL (2005). A comparison of experience-dependent plasticity in the visual and somatosensory systems.. Neuron.

[3] Chang EF, Merzenich MM (2003). Environmental noise retards auditory cortical development.. Science.

[4] Hudson R (1999). From molecule to mind: the role of experience in shaping olfactory function.. J Comp Physiol A.

[5] Oboti L, Savalli G, Giachino C, De Marchis S, Panzica GC (2009). Integration and sensory experience-dependent survival of newly generated neurons in the accessory olfactory bulb of female mice.. Eur J Neurosci.

[6] Pinsker HM, Hening WA, Carew TJ, Kandel ER (1973). Long-term sensitization of a defensive withdrawal reflex in *Aplysia*.. Science.

[7] Duerr JS, Quinn WG (1982). Three Drosophila mutations that block associative learning also affect habituation and sensitization.. Proc Natl Acad Sci U S A.

[8] Monteith LG (1963). Habituation and Associative Learning in *Drino bohemica* Mesn. (Diptera: Tachinidae).. Can Entomol.

[9] Braun G, Bicker G (1992). Habituation of an appetitive reflex in the honeybee.. J Neurophysiol.

[10] Grubb MS, Thompson ID (2004). The influence of early experience on the development of sensory systems.. Curr Opin Neurobiol.

[11] Walters E, Illich P, Weeks J, Lewin M (2001). Defensive responses of larval *Manduca sexta* and their sensitization by noxious stimuli in the laboratory and field.. J Exp Biol.

[12] Rakitin A, Tomsic D, Maldonado H (1991). Habituation and sensitization to an electrical shock in the crab *Chasmagnathus*. Effect of background illumination.. Physiol Behav.

[13] Anderson P, Sadek MM, Hansson BS (2003). Pre-exposure modulates attraction to sex pheromone in a moth.. Chem Senses.

[14] Menzel R, Muller U (1996). Learning and memory in honeybees: from behavior to neural substrates.. Annu Rev Neurosci.

[15] Heisenberg M, Borst A, Wagner S, Byers D (1985). Drosophila mushroom body mutants are deficient in olfactory learning.. J Neurogenet.

[16] Zhou DS, Wang CZ, van Loon JJA (2009). Chemosensory basis of behavioural plasticity in response to deterrent plant chemicals in the larva of the small cabbage white butterfly *Pieris rapae*.. J Insect Physiol.

[17] Davis EE (1984). Regulation of sensitivity in the peripheral chemoreceptor systems for host-seeking behaviour by a haemolymph-borne factor in *Aedes aegypti*.. J Insect Physiol.

[18] Silvegren G, Löfstedt C, Qi Rosén W (2005). Circadian mating activity and effect of pheromone pre-exposure on pheromone response rhythms in the moth *Spodoptera littoralis*.. J Insect Physiol.

[19] Anderson P, Hansson BS, Nilsson U, Han Q, Sjoholm M (2007). Increased behavioral and neuronal sensitivity to sex pheromone after brief odor experience in a moth.. Chem Senses.

[20] Barrozo RB, Gadenne C, Anton S (2010). Switching attraction to inhibition: mating-induced reversed role of sex pheromone in an insect.. J Exp Biol.

[21] Gabel B (1992). Tansy flowers attract european grapevine moth females, *Lobesia botrana* Den. and Schiff. (Lep., Tortricidae).. J Appl Entomol.

[22] Haynes KF, Zhao JZ, Latif A (1991). Identification of floral compounds from *Abelia grandiflora* that stimulate upwind flight in cabbage looper moths.. J Chem Ecol.

[23] Hansson BS, Christensen TA, Hansson BS (1999). Functional characteristics of the antennal lobe.. Insect Olfaction.

[24] Reddy GVP, Guerrero A (2004). Interactions of insect pheromones and plant semiochemicals.. Trends Plant Sci.

[25] Chapman RF (1974). The chemical inhibition of feeding by phytophagous insects: a review.. Bull Entomol Res.

[26] Jorgensen K, Almaas TJ, Marion-Poll F, Mustaparta H (2007). Electrophysiological characterization of responses from gustatory receptor neurons of sensilla chaetica in the moth *Heliothis virescens*.. Chem Senses.

[27] Fan RJ, Anderson P, Hansson BS (1997). Behavioural analysis of olfactory conditioning in the moth *Spodoptera littoralis* (Boisd.) (Lepidoptera: noctuidae).. J Exp Biol.

[28] Hartlieb E, Anderson P, Hansson BS (1999). Appetitive learning of odours with different behavioural meaning in moths.. Physiol Behav.

[29] Le Bourg E (1996). Hypergravity and aging in *Drosophila melanogaster*. 8. Proboscis-extension-response threshold to sucrose.. Gerontology.

[30] Bitterman ME, Menzel R, Fietz A, Schäfer S (1983). Classical conditioning of proboscis extension in honeybees (*Apis mellifera*).. J Comp Physiol A.

[31] Dethier VG, Bowdan E (1989). The effect of alkaloids on sugar receptors and the feeding behaviour of the blowfly.. Physiol Entomol.

[32] Amakawa T (2001). Effects of age and blood sugar levels on the proboscis extension of the blow fly *Phormia regina*.. J Insect Physiol.

[33] Anton S, Evengaard K, Barrozo RB, Anderson P, Skals N (2011). Brief predator sound exposure elicits behavioral and neuronal long-term sensitization in the olfactory system of an insect.. Proc Nat Acad Sci U S A.

[34] Kramer E (1992). Attractivity of pheromone surpassed by time-patterned application of two nonpheromone compounds.. J Insect Behav.

[35] Sakuma M (2002). Virtual reality experiments on a digital servosphere: guiding male silkworm moths to a virtual odour source.. Comput Electron Agr.

[36] Hartlieb E, Rembold H (1996). Behavioral response of female *Helicoverpa (Heliothis) armigera* HB. (Lepidoptera: Noctuidae) moths to synthetic pigeonpea (*Cajanus cajan* L.) kairomone.. J Chem Ecol.

[37] Ramaswamy SB, Cohen NE, Hanson FE (1992). Deterrence of feeding and oviposition responses of adult *Heliothis virescens* by some compounds bitter-tasting to humans.. Entomol Exp Appl.

[38] Jorgensen K, Stranden M, Sandoz JC, Menzel R, Mustaparta H (2007). Effects of two bitter substances on olfactory conditioning in the moth *Heliothis virescens*.. J Exp Biol.

[39] Kvello P, Jorgensen K, Mustaparta H (2010). Central gustatory neurons integrate taste quality information from four appendages in the moth *Heliothis virescens*.. J Neurophysiol.

[40] Hammer M, Braun G, Mauelshagen J (1994). Food-induced arousal and nonassociative learning in honeybees: dependence of sensitization on the application site and duration of food stimulation.. Behav Neural Biol.

[41] Diaz-Cenzano E, Chotro MG (2010). The effect of taste familiarity on intake and taste reactivity in infant rats.. Dev Psychobiol.

[42] Gonzalez KM, Peo C, Livdahl T, Kennedy LM (2008). Experience-induced changes in sugar taste discrimination.. Chem Senses.

[43] Homberg U, Müller U, Hansson BS (1999). Neuroactive substances in the antennal lobe.. Insect Olfaction.

[44] Nässel D, Homberg U (2006). Neuropeptides in interneurons of the insect brain.. Cell Tissue Res.

[45] Niven JE, Laughlin SB (2008). Energy limitation as a selective pressure on the evolution of sensory systems.. J Exp Biol.

[46] Prados J, Redhead ES (2002). Preexposure effects in spatial learning: from gestaltic to associative and attentional cognitive maps.. Psicologica.

[47] Morris RGM (1981). Spatial localization does not require the presence of local cues.. Learn Motiv.

[48] O'Keefe J, Nadel L (1979). The hippocampus as a cognitive map.. Behav Brain Sci.

[49] Tolman EC (1948). Cognitive maps in rats and men.. Psychol Review.

[50] Party V, Hanot C, Said I, Rochat D, Renou M (2009). Plant terpenes affect intensity and temporal parameters of pheromone detection in a moth.. Chem Senses.

[51] Popescu A (2008). Mechanisms of gustatory coding in *Spodoptera littoralis*..

[52] Rouyar A, Party V, Prešern J, Blejec A, Renou M (2011). A general odorant background affects the coding of pheromone stimulus intermittency in specialist olfactory receptor neurones.. PLoS ONE.

[53] Zar JH, Mc Elroy WD, Swanson CP (1999). Biostatistical analysis, 4^th^ edition..

[54] Christensen TA, Hildebrand JG (1987). Male-specific, sex pheromone-selective projection neurons in the antennal lobes of the moth *Manduca sexta*.. J Comp Physiol A.

[55] Hodgson E, Lettvin J, Roeder K (1955). Physiology of a primary chemoreceptor unit.. Science.

[56] Marion-Poll F, Pers J (1996). Un-filtered recordings from insect taste sensilla.. Entomol Exp Appl.

